# Progress in the Construction of General Appliances

**Published:** 1920-05-22

**Authors:** 


### Progress in the Construction of General Appliances.

A practical demonstration of orthopædic appliances recently given at the Welbeck Palace Hotel by the Gloucestershire Surgical Appliances Co., of Belle Vue Lawn, Cheltenham, and Woodstock House, 10 and 12 James Street, Manchester Square, W. 1, is of special interest as indicating the power of ameliorating the consequences of damage to joints and limbs resulting from disease and injury by the application of skilfully made appliances. There is practically no limit to the possibilities in this direction by the combination of surgical and mechanical skill with inventive genius.

We were particularly interested in the design of an excised elbow support. This appliance consists of hollow-steel side supports jointed at the elbow to which are attached springs with adjustable tension. It is fixed to the arm by means of laced sockets arranged above and below the elbow, and additional support is provided by shoulder straps attached to the latter. As the instrument is very light and well finished and the sockets properly ventilated, discomfort to the patient is reduced to a minimum. The makers claim that by the aid of this artificial fulcrum the wasted muscles are much improved and the limb rendered serviceable.

The appliances exhibited by this company for correcting deformities resulting from infantile paralysis showed considerable mechanical genius, but what is of greater importance, there was evidence that those responsible for their manufacture had the ability to adapt ingenious contrivances to meet individual needs and the requirements of the orthopædic surgeon.

Among other specialities we inspected a jacket made of "Duralium" (probably an aluminium alloy). This material appears to be more suitable than poroplastic felting, which has a tendency to mould itself to the deformity rather than to correct it. The jacket is well ventilated and very light.

